# Determinants of the decline in mortality from acute stroke in England: linked national database study of 795 869 adults

**DOI:** 10.1136/bmj.l1778

**Published:** 2019-05-22

**Authors:** Olena O Seminog, Peter Scarborough, F Lucy Wright, Mike Rayner, Michael J Goldacre

**Affiliations:** 1 Unit of Health-Care Epidemiology, Big Data Institute, Nuffield Department of Population Health, NIHR Oxford Biomedical Research Centre, University of Oxford, Oxford OX3 7LF, UK; 2 Centre on Population Approaches for Non-communicable Disease Prevention, Nuffield Department of Population Health, NIHR Biomedical Research Centre at Oxford, University of Oxford, Oxford, UK

## Abstract

**Objectives:**

To study trends in stroke mortality rates, event rates, and case fatality, and to explain the extent to which the reduction in stroke mortality rates was influenced by changes in stroke event rates or case fatality.

**Design:**

Population based study.

**Setting:**

Person linked routine hospital and mortality data, England.

**Participants:**

795 869 adults aged 20 and older who were admitted to hospital with acute stroke or died from stroke.

**Main outcome measures:**

Stroke mortality rates, stroke event rates (stroke admission or stroke death without admission), and case fatality within 30 days after stroke.

**Results:**

Between 2001 and 2010 stroke mortality rates decreased by 55%, stroke event rates by 20%, and case fatality by 40%. The study population included 358 599 (45%) men and 437 270 (55%) women. Average annual change in mortality rate was −6.0% (95% confidence interval −6.2% to −5.8%) in men and −6.1% (−6.3% to −6.0%) in women, in stroke event rate was −1.3% (−1.4% to −1.2%) in men and −2.1% (−2.2 to −2.0) in women, and in case fatality was −4.7% (−4.9% to −4.5%) in men and −4.4% (−4.5% to −4.2%) in women. Mortality and case fatality but not event rate declined in all age groups: the stroke event rate decreased in older people but increased by 2% each year in adults aged 35 to 54 years. Of the total decline in mortality rates, 71% was attributed to the decline in case fatality (78% in men and 66% in women) and the remainder to the reduction in stroke event rates. The contribution of the two factors varied between age groups. Whereas the reduction in mortality rates in people younger than 55 years was due to the reduction in case fatality, in the oldest age group (≥85 years) reductions in case fatality and event rates contributed nearly equally.

**Conclusions:**

Declines in case fatality, probably driven by improvements in stroke care, contributed more than declines in event rates to the overall reduction in stroke mortality. Mortality reduction in men and women younger than 55 was solely a result of a decrease in case fatality, whereas stroke event rates increased in the age group 35 to 54 years. The increase in stroke event rates in young adults is a concern. This suggests that stroke prevention needs to be strengthened to reduce the occurrence of stroke in people younger than 55 years.

## Introduction

Stroke mortality rates have been declining in almost every country.^1^ Reduction in mortality could result from a decline in disease occurrence or a decline in case fatality, or both. A reduction in stroke event rates could result from better management of risk factors, achieved through lifestyle modification and prevention. From a public health perspective, decline in disease is preferable to decline in case fatality, because people who survive a stroke have high rates of disability and an increased risk of developing vascular dementia.^2^ For patients after stroke and their families, however, quality of care is paramount to increase the chance of survival. Improved case fatality at 30 days after stroke is almost certainly a result of improvements in treatment and management, and perhaps in prevention, which could reduce the severity of strokes.^3^ ^4^

Within existing evidence, it is not completely understood which of the two factors—declining event rates or declining case fatality—has a more important role in the observed reduction in mortality rates from stroke in England. A study of acute myocardial infarction reported that a decline in event rates contributed just over a half and improved survival at 30 days just under a half to the decline in mortality.^5^ No studies analysed the factors that contributed to the decline in mortality from acute stroke. Data from clinical trials or biobank studies alone cannot be used to find the answer because they do not cover whole, representative populations. England, however, has a large national linked dataset of electronic hospital records and mortality statistics, which is well suited for such studies.

We quantified the contribution of changes in stroke event rates and case fatality to the reduction in stroke mortality using methods developed by the World Health Organization’s MONICA (monitoring of trends and determinants in cardiovascular disease) study,^6^ ^7^ as was used in a similar study of myocardial infarction.^5^ We report on temporal changes in age specific stroke mortality rates, event rates, and case fatality.

## Methods

### Data sources

Data were obtained from two national datasets of routine data, hospital episode statistics (HES) and national mortality statistics. The HES data were supplied by Health and Social Care Information Centre (renamed as NHS Digital). The Office for National Statistics supplied the mortality data. The linkage of records between the datasets was based on encrypted personal identifiers, including National Health Service number, date of birth, and postcode, sent in encrypted form by the data providers to the Unit of Health-Care Epidemiology, University of Oxford, where the linkage was done. The database covers the whole of England and contains information on every stroke that resulted in hospital admission to an NHS hospital or in a death without hospital admission. The NHS funds most of the hospital care in England. HES also receives information on private patients (although a minority of emergency admissions in England) managed in NHS hospitals. Thus, the database provides nearly complete coverage, except for private hospitals, of all patients admitted to hospital for stroke events in England. From national mortality data held by NHS Digital at https://digital.nhs.uk/data-and-information we obtained information on all deaths certified as death from stroke, including those that occurred out of hospital or in an emergency department, before a patient was admitted to a ward.

### Study population and selection criteria

In the analysis we included all residents of England aged 20 and older who were admitted to hospital with stroke or died from stroke between 1 January 2001 and 31 December 2010. We defined population based mortality for stroke as a death with stroke as the certified underlying cause of death, and this was expressed per 100 000 population of England.

Following the terminology used by the MONICA study, we defined the occurrence of stroke as events,^7^ defined as hospital admission for stroke, or a death with stroke as the underlying cause without a corresponding hospital admission for stroke in the preceding 30 days. If patients had more than one stroke, we included multiple events in the analysis if these events were more than 30 days apart. Event rates were expressed per 100 000 resident population of England. Case fatality was defined as the proportion of events that were fatal within 30 days after stroke, and this was expressed as a percentage.

Strokes were selected using the ICD-10 (international classification of diseases, 10th revision) codes I61-I64 as the primary diagnosis on a hospital record or as the certified underlying cause of death. We restricted analysis to emergency admissions and hospital transfers and excluded elective admissions. Only patients who spent more than a day in hospital and who were discharged alive were included in the analysis. The length of stay criterion was not applied to patients who had a stroke recorded as the principal diagnosis and who died in hospital: these cases were included. In patients who were discharged home when the diagnosis was not confirmed, we used the one day criterion to exclude cases of suspected acute stroke, which likely were transient ischaemic attacks or similar.

### Statistical analysis

#### Calculating mortality rate, event rate, and case fatality

We calculated stroke mortality rates, event rates, and 30 day case fatality in men and women in individual calendar years. The corresponding 95% confidence intervals were calculated assuming a Poisson distribution. Mortality rates were calculated by dividing the number of stroke deaths in a calendar year by mid-year resident populations; the population counts were obtained from the Office for National Statistics website. Our measure of stroke occurrence was a stroke event, and to calculate annual rates we divided the number of stroke events—both hospital admissions and deaths—by mid-year populations and expressed these per 100 000 population. Mortality and event rates in men and women as population based rates were directly age standardised to the 2013 European standard population. Case fatality was calculated by dividing the total number of deaths that occurred within 30 days after hospital admission for stroke, and included out-of-hospital deaths, by the total number of stroke events, multiplied by 100 and expressed as a percentage. Case fatality was directly age standardised in five year age groups using the combined 10 year study population as the standard population. In addition, we standardised case fatality to the 2013 European standard population to enable comparison with other studies.

#### Analysis of trends in stroke mortality rates, event rates, and case fatality

The estimate of change in rates was the average annual percentage change. For each measure—mortality rate, stroke event rate, and case fatality—we ran a separate regression model for all ages combined and for specified age groups. Age specific analysis was performed in six age groups: 20-34, 35-54, 55-64, 65-74, 75-84, and 85 years and older. To allow comparison with the MONICA results, we repeated the same analysis restricting the study cohort to people younger than 65 years.

We used a Poisson regression model to calculate the average annual percentage change in mortality rates and event rates. In the analysis of changes in stroke mortality rates, the dependent variable was stroke deaths. In the analysis of changes in stroke event rates, the dependent variable was stroke events. In both analyses the calendar year of admission was an independent variable and, because we did Poisson regression analysis for rates, we used the corresponding age specific mid-year population as the exposure variable.

Two methods were used to calculate average annual changes in case fatality. Using the dataset, we calculated the annual changes in case fatality in a generalised linear model with binominal distribution, with death within 30 days after stroke as the dependent variable. We also calculated the annual change in case fatality as a difference in annual change in mortality rates and stroke event rates using the WHO MONICA formula by subtracting the event rate from the mortality rate.

#### Calculating determinants of the decline in stroke mortality rates

We estimated the relative contribution of changes in event rates and case fatality to changes in stroke mortality over years using an equation from the WHO MONICA study, which states that ∆M=∆C+∆E, where ∆M is the annual percentage change in mortality rate, ∆C is the annual percentage change in case fatality, and ∆E is the annual percentage change in event rate.^6^

The equation is derived: if M is the mortality rate, E is the event rate, and C is the case fatality, then at any moment M=E×C, since the mortality rate is just the event rate multiplied by the case fatality. To estimate the change in mortality rates by time (t), differentiation (d) is used, whereby it follows that dM/dt=E×dC/dt+C×dE/dt, or M’=E×C’+C×E’ where M’=dM/dt and similar for E and C. The annual percentage change in rates is simply the annual change divided by the rate, so that ΔM=M’/M, ΔE=E’/E and ΔC=C’/C. From this, ΔM=M’/M=(E×C’+C×E’)/(E×C)=C’/C+E’/E=ΔC+ΔE.

Using this formula, we separated the contribution of percentage change in stroke event rates and case fatality to the percentage change in mortality rates. The relative contribution of each of the two parameters in the formula is calculated as the percentage of total mortality, which is set to 100%.

### Patient and public involvement

The investigation did not conduct any interaction or intervention with participants on whom data were obtained. Patients and the public were not involved in the design, analysis, or interpretation of this study. The analysis was done on anonymised data, and therefore we are not able to consult with or disseminate our findings to participants.

## Results

The linked hospital episode and mortality dataset comprised data on 947 497 stroke events, including 337 085 stroke deaths, in 795 869 people. Of these events, 521 788 (55.1%) of strokes and 207 198 (61.5%) of stroke deaths occurred in women (table 1). Although between 2001 and 2010 there was no change in the total number of stroke events in men and a modest reduction in women, the total number of stroke deaths decreased in men, from 15 904 in 2001 to 10 481 in 2010, and in women from 25 947 to 16 117. The mean age at the onset of stroke was 72 years (SD 13 years) in men and 76 (SD 14) years in women. The mean age of those who died from stroke was 79 (SD 11) years in men and 83 (SD 9) years in women.

**Table 1:** Characteristics of acute stroke events and deaths by sex and calendar period, England

Characteristics	2001-10*			2001			2010	
	Men (n=358 599)	Women (n=437 270)		Men (n=36 477)	Women (n=47 318)		Men (n=36 957)	Women (n=42 644)
**Stroke events**								
Mean (SD) age (years)	72 (13)	76 (14)		71 (13)	75 (14)		73 (13)	77 (14)
Age group (years):								
20-34	3040	2525		308	231		304	248
35-54	31 300	19 027		2770	1713		3597	2237
55-64	52 409	28 449		4898	2791		5640	3045
65-74	100 942	74 429		11 088	8308		9900	7184
75-84	153 201	189 492		15 833	20 719		14 513	16 760
≥85	84 817	207 866		8234	21 758		9495	20 944
Total No of stroke events	425 709	521 788		43 131	55 520		43 449	50 418
Crude event rate (95% CI) per 100 000†	229.6 (228.9 to 230.2)	263.7 (263.0 to 264.4)		241.6 (239.3 to 243.9)	288.1 (285.7 to 290.5)		224.0 (221.9 to 226.2)	246.9 (244.7 to 249.0)
Age adjusted event rate (95% CI) per 100 000‡	309.8 (308.8 to 310.7)	254.1 (253.4 to 254.8)		345.1 (341.7 to 348.5)	280.2 (277.9 to 282.5)		284.5 (281.8 to 287.2)	233.7 (231.7 to 235.8)
**Stroke deaths**								
Mean (SD) age (years)	79 (11)	83 (9)		78(10)	83 (9)		79 (11)	84 (9)
Age group (years):								
20-34	314	222		37	22		26	12
35-54	4108	2515		436	293		363	201
55-64	8214	5008		964	582		680	422
65-74	22 955	18 308		3151	2578		1765	1353
75-84	53 281	70 758		6647	9109		3829	4867
≥85	41 015	110 387		4669	13 363		3818	9262
Total No of stroke deaths	129 887	207 198		15 904	25 947		10 481	16 117
Crude mortality rate (95% CI) per 100 000†	70.0 (69.7to 70.4)	104.7 (104.3 to 105.2)		89.10 (87.7 to 90.5)	134.70 (133.0 to 136.3)		54.0 (53.0 to 55.1)	78.90 (77.7 to 80.1)
Age standardised mortality rate (95% CI) per 100 000‡	103.1 (102.5 to 103.6)	98.0 (97.6 to 98.5)		139.60 (137.3 to 141.9)	127.90 (126.3 to 129.5)		73.90 (72.5 to 75.4)	71.70 (70.6 to 72.8)
**30 day deaths after stroke**								
Mean (SD) age (years)	78 (11)	83 (10)		78 (11)	83 (9)		78 (12)	84 (10)
Age group (years):								
20-34	450	310		51	33		34	23
35-54	4771	3011		510	338		413	256
55-64	8715	5468		1028	653		705	457
65-74	21 910	17 930		3028	2523		1697	1294
75-84	48 252	64 064		6188	8466		3390	4334
≥85	36 550	99 264		4333	12 443		3256	8015
Total No of 30 day case fatalities	120 648	190 047		15 138	24 456		9495	14 379
Crude case fatality (%)	28.3	36.2		35.1	44.0		21.9	28.5
Age standardised case fatality (%)‡	33.9	36.4		41.8	44.1		26.4	28.5

* Mean ages, total counts of stroke events, and deaths in each sex and age group, as well as crude and age standardised rates, were calculated for a combined 10 year period.

† Population denominators were obtained from the Office for National Statistics website.

‡ Mortality rates and stroke event rates for all ages were age standardised to the 2013 European standard population (see Methods), and case fatality was standardised to the study population.

### Trends over time in mortality rates

**Fig 1:**
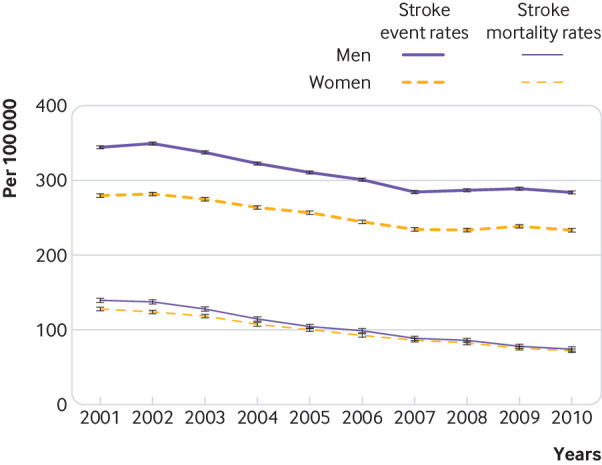
Trends in age standardised stroke event rates and stroke mortality rates in men and women between 2001 and 2010, England

The age standardised mortality rates decreased by 55% during the study period, and a reduction was observed in all age groups. Age standardised mortality rates in men decreased from 140 (95% confidence interval 137 to 142) per 100 000 population in 2001 to 74 (73 to 75) per 100 000 in 2010 and in women from 128 (126 to 130) per 100 000 in 2001 to 72 (71 to 73) per 100 000 in 2010 (fig 1). The annual change in mortality rates in men was –6.0% (95% confidence interval –6.2% to –5.8%) and in women was –6.1% (–6.3% to –6.0%). The largest reduction in mortality rates was in men and women aged 65 to 74, with an annual change in men of –8.1% (–8.6% to –7.7%) and in women of –8.3% (–8.7% to –7.8%). The lowest average annual reduction in mortality rates was in the youngest age group, 20-34 years: –4.1% (–7.8% to –0.4%) in men and –4.5% (–8.8% to 0.0%) in women.

### Trends over time in stroke event rates

Between 2001 and 2010 stroke event rates in men decreased from 345 (342 to 349) per 100 000 population to 285 (282 to 287) per 100 000, and in women from 280 (278 to 283) per 100 000 to 234 (232 to 236) per 100 000 (fig 1), with an average annual reduction in men of –1.3% (–1.4% to –1.2%) and in women of –2.1% (–2.2% to –2.0%). The reduction in stroke event rate was larger in older age groups: in men and women aged 85 and older, for example, it was –3.4% (–3.6% to –3.1%) and –2.7% (–2.8% to –2.5%), respectively (table 2). In contrast, stroke event rates among people aged 35-54 years increased by 2% each year. No statistically significant change was observed in men and women younger than 35 years.

**Table 2:** Age specific rates and average annual percentage change in stroke mortality, stroke events, and case fatality in men and women between 2001 and 2010, England

Age group (years)	Mortality rate per 100 000 (95% CI)				Event rate per 100 000 (95%CI)				Case fatality (%)					Contribution to mortality decline (%)	
	2001	2010	Annual % change (95% CI)		2001	2010	Annual % change (95% CI)		2001	2010	Estimated % change, MONICA formula	Annual % change, from dataset (95% CI)		Event rate	Case fatality
Men:															
20-34	0.73 (0.51 to 1.01)	0.49 (0.32 to 0.71)	−4.1 (−7.8 to −0.4)		6.07 (5.4 to 6.8)	5.68 (5.06 to 6.35)	0.1 (−1.1 to 1.4)		16.6 (12.3 to 21.8)	11.2 (7.7 to 15.6)	−4.3	−3.4 (−6.3 to −0.5)		0	100
35-54	6.32 (5.74 to 6.94)	5.01 (4.51 to 5.55)	−3.8 (−4.8 to −2.8)		40.1 (38.7 to 41.7)	49.7 (48.0 to 51.3)	2.2 (1.8 to 2.6)		18.4 (16.8 to 20.1)	11.5 (10.4 to 12.6)	−6.0	−5.3 (−6.2 to −4.5)		0	100
55-64	37.8 (35.1 to 39.8)	22.7 (21.0 to 24.5)	−6.1 (−6.8 to −5.3)		189.87 (184.6 to 195.3)	188.3 (183.4 to 193.2)	−0.6 (−0.9 to −0.3)		21.0 (19.7 to 22.3)	12.5 (11.6 to 13.5)	−5.5	−4.9 (−5.5 to −4.2)		9.6	90.4
65-74	163.4 (157.8 to 169.3)	82.37 (78.6 to 86.3)	−8.1 (−8.6 to −7.7)		575.1 (564.5 to 585.9)	462.0 (453.0 to 471.2)	−2.7 (−2.9 to −2.5)		27.3 (26.3 to 28.3)	17.1 (16.3 to 18.0)	−5.4	−5.0 (−5.4 to −4.6)		33.3	66.7
75-84	602.8 (588.9 to 617.5)	303.3 (293.8 to 313.0)	−7.7 (−8.0 to −7.4)		1435.8 (1413.6 to 1458.4)	1149.5 (1130.9 to 1168.4)	−2.9 (−3.1 to −2.8)		39.1 (38.1 to 40.1)	23.4 (22.6 to 24.2)	−4.8	−5.0 (−5.2 to −4.7)		37.9	62.1
≥85	1748.7 (1698.9 to 1799.6)	971.5 (940.9 to 1002.8)	−7.0 (−7.4 to −6.7)		3083.9 (3017.6 to 3151.2)	2416.0 (2367.7 to 2465.1)	−3.4 (−3.6 to −3.1)		52.5 (51.1 to 54.2)	34.3 (33.1 to 35.5)	−3.7	−4.3 (−4.5 to −4.2)		47.8	52.2
All <65	11.4 (10.8 to 12.0)	7.3 (6.9 to 7.8)	−5.3 (−5.9 to −4.7)		62.3 (60.9 to 63.7)	65.4 (64.1 to 66.7)	0.8 (0.6 to 1.0)		19.8 (19.0 to 20.7)	12.0 (11.4 to 12.7)	−4.5	−5.0 (−5.5 to −4.5)		0	100
All ages*	139.6 (137.3 to 141.9)	73.9 (72.5 to 75.4)	−6.0 (−6.2 to −5.8)		345.1 (341.7 to 348.5)	284.5 (281.8 to 287.2)	−1.3 (−1.4 to −1.2)		41.8 (41.3 to 42.4)	26.4 (25.9 to 26.9)	−4.7	−4.7 (−4.9 to −4.5)		21.7	78.3
Women:															
20-34	0.43 (0.27 to 0.66)	0.23 (0.12 to 0.41)	−4.5 (−8.8 to 0.0)		4.54 (3.98 to 5.17)	4.82 (4.24 to 5.46)	0.7 (−0.7 to 2.1)		14.3 (9.8 to 20.1)	9.3 (5.9 to 13.9)	−5.2	−5.1 (−8.6 to −1.5)		0	100
35-54	4.19 (3.72 to 4.69)	2.7 (2.37 to 3.14)	−5.3 (−6.6 to −4.0)		24.5 (23.3 to 25.7)	30.4 (29.3 to 31.7)	2.1 (1.6 to 2.6)		19.7 (17.7 to 22.0)	11.4 (10.1 to 12.9)	−7.5	−5.6 (−6.7 to −4.5)		0	100
55-64	22.0 (20.2 to 23.8)	13.6 (12.3 to 14.9)	−5.7 (−6.6 to −4.8)		105.4 (101.5 to 109.4)	97.8 (94.3 to 101.3)	−0.8 (−1.2 to −0.4)		23.4 (21.6 to 25.3)	15.0 (13.7 to 16.4)	−4.9	−4.2 (−5.0 to −3.4)		13.6	86.4
65-74	119.0 (113.5 to 122.6)	57.7 (54.7 to 60.9)	−8.3 (−8.7 to −7.8)		380.2 (372.1to 388.5)	306.5 (299.4 to 313.6)	−2.9 (−3.1 to −2.6)		30.4 (29.2 to 31.6)	18.0 (17.0 to 19.0)	−5.4	−5.0 (−5.4 to −4.6)		34.5	65.5
75-84	548.4 (537.2 to 559.8)	293.4 (285.2 to 301.7)	−7.0 (−7.2 to −6.7)		1247.5 (1230.5 to 1264.6)	1010.3 (995.0 to 1025.7)	−2.7 (−2.9 to −2.6)		40.9 (40.0 to 41.7)	25.9 (25.1 to 26.6)	−4.3	−4.5 (−4.7 to −4.3)		39.0	61.0
≥85	1931.4 (1898.7 to 1964.4)	1150.8 (1127.5 to 1174.5)	−6.2 (−6.4 to −6.0)		3144.7 (3103.0 to 3186.7)	2602.4 (2567.3 to 2637.9)	−2.7 (−2.8 to −2.5)		57.2 (56.2 to 58.2)	38.3 (37.4 to 39.1)	−3.5	−4.3 (−4.5 to −4.2)		43.3	56.7
All <65	6.9 (6.5 to 7.4)	4.3 (3.9 to 4.6)	−5.0 (−5.7 to −4.3)		36.0 (35.0 to 37.0)	36.6 (35.7 to 37.6)	0.8 (0.5 to 1.2)		21.5 (20.3 to 22.6)	13.4 (12.5 to 14.3)	−4.2	−4.8 (−5.4 to −4.2)		0	100
All ages*	127.9 (126.3 to 129.5)	71.7 (70.6 to 72.8)	−6.1 (−6.3 to −6.0)		280.2 (277.9 to 282.5)	233.7 (231.7 to 235.8)	−2.1 (−2.2 to −2.0)		44.1 (43.7 to 44.5)	28.5 (28.1 to 28.9)	−4.0	−4.4 (−4.5 to −4.2)		34.4	65.6

* Mortality rates and stroke event rates for all ages were age standardised to the 2013 European standard population (see Methods), and case fatalities were standardised to the study population.

### Trends over time in case fatality

**Fig 2:**
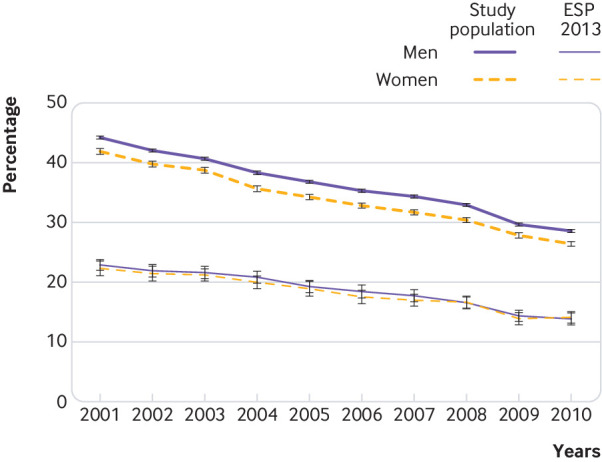
Trends in 30 day stroke case fatality, including in-hospital and out-of-hospital deaths, age standardised to study population and to 2013 European standard population (ESP), in men and women between 2001 and 2010, England

Case fatality at 30 days decreased by about 40% between 2001 and 2010 (fig 2). A choice of the standard population made a noticeable difference to the absolute values of age standardised case fatality; for example, in 2001 case fatality in men was 42% when age standardised to the study population and 22% when age standardised to the 2013 European standard population. Given the large difference in case fatality introduced by using the standard population, figure 2 presents the two sets of results alongside each other.

Analysis of age specific case fatality showed a substantial reduction in 30 day mortality after stroke in all age groups. Case fatality after stroke was the highest in older age groups. In 2001 more than half of all patients with stroke aged 85 years and older died within 30 days; within 10 years it decreased to 34% (33% to 36%) in men and 38% (37% to 39%) in women (table 2). Average annual changes in case fatality were calculated using the WHO MONICA formula and regression analysis. Each method produced similar results. On average the annual reduction in case fatality was 4.7% in men and 4.0% in women calculated using the WHO MONICA formula, and 4.7% (4.9% to 4.5%) in men and 4.4% (4.5% to 4.2%) in women calculated in regression analysis. When trends were analysed by age group, the largest average annual reduction in case fatality was observed in men and women aged 35-54 years, at 6.0% and 7.5% each year, respectively.

### Determinants of the reduction in mortality rates

**Fig 3:**
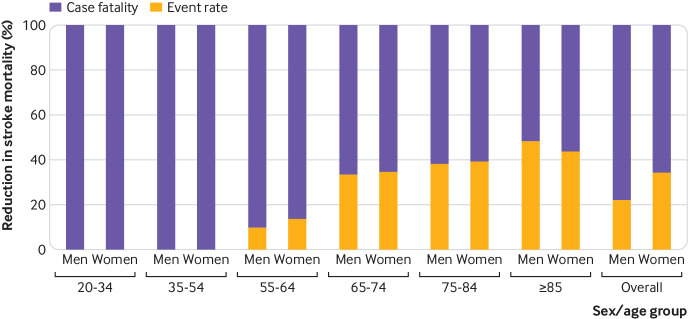
Percentage contribution of changes in stroke case fatality and event rates to percentage reduction in stroke mortality by age group in men and women between 2001 and 2010, England

Table 2 and figure 3 show the contribution to reduction in mortality rates of changes in event rates and case fatality for men and women of all ages and in specific age groups. Seventy one per cent of the reduction in mortality from stroke overall was attributable to the reduction in case fatality and 29% to the reduction in stroke event rates. In men and women, the equivalent percentage contributions were, respectively, 78% and 66% and 22% and 34%. The contribution of these two factors varied between the age groups. In the two youngest age groups, 20-34 and 35-54 years, the decline in stroke mortality was attributable to a reduction in case fatality. A gradual increase occurred in the relative contribution of changes in stroke event rates to mortality reduction with increasing age, from 10% in men and 14% in women aged 55-64 years, to 48% in men and 43% in women aged 85 years and older.

## Discussion

During the first decade of the 21st century stroke mortality rates in England halved, stroke event rates decreased by about 20%, and case fatality decreased by about 40%. Most of the decline in stroke mortality rates—78% in men and 66% in women—resulted from a reduction in case fatality, and the remaining 22% and 34%, respectively, from a reduction in event rates. Important variations existed between young and old people: in those younger than 55 years, the reduction in mortality from stroke was attributed to improved survival, and in those aged 85 years or older, improved survival and reduction in event rates were equally important for mortality reduction. Stroke event rates increased in people aged 35-54 years, on average by 2% each year, which contrasts with the downward trend observed in the other age groups. In this age group, however, the increase in event rates did not translate into an increase in mortality rates, as it was offset by the reduction in case fatality.

The burden of stroke in England is decreasing, when 2010 is compared with 2001, as reflected by the reduction in absolute numbers of stroke deaths and in the stable absolute numbers of stroke events in men and the reduction in women.

### Strengths and limitations of this study

This large study of stroke events and mortality in England includes all patients admitted to an NHS hospital and all deaths in England, and it covers a continuous period of 10 calendar years. The findings are applicable to the whole of England. However, epidemiological and medical care factors vary from place to place, and a challenge for local investigators and those in other countries is to determine how these compare with profiles of mortality, event rates, and case fatality in their settings. The study is free from selection biases that might arise when data collected by stroke registries are analysed, which are limited in terms of populations and period covered, or clinical trials in which participants are selected and therefore might not be representative of all patients with stroke. The large size of the study population provided the statistical power to undertake age specific analyses. Unlike other studies such as MONICA, our study cohort did not have an upper age limit, and we reported trends in old as well as young adults.^6^ ^7^

We relied on the quality of stroke diagnoses in routine hospital statistics and mortality statistics. Validation studies have consistently reported more than 90% accuracy of stroke codes in linked HES datasets.^8^ ^9^ ^10^ For this study we combined all strokes with ICD-10 codes I61 to I64. We did not analyse haemorrhagic and ischaemic stroke separately, because additional analyses (not shown) showed that the reporting of stroke type in linked HES has been inconsistent through the study period.^11^ For example, in 2001 the type of stroke was not recorded for about half of all hospital admissions for stroke, and this decreased to less than 20% in 2010. Therefore, examining trends in rates reported for stroke types, as distinct from stroke overall, could be misleading as they are likely to be affected by improved recording of stroke type rather than true changes.

Changes in stroke event rates could be subject to improved sensitivity of stroke diagnostics, including better quality of brain imaging, as well as increasing public awareness of the signs of stroke. Although these might have resulted in an increase in hospital admissions, particularly for milder strokes, we report a decrease in stroke event rates.

In calculating event rates, we only included people who were admitted to hospital with acute stroke or those who died from stroke. Our study would not capture any silent infarcts or small strokes that did not result in a hospital admission or death, and therefore this study might have missed cases of stroke that were treated wholly without hospital admission, such as patients managed in nursing homes.

Information on stroke severity is not recorded in routine hospital statistics. Some of the decline in case fatality reported here could be a result of a reduction in stroke severity and an increase in hospital admissions for less severe or suspected strokes, rather than advances in acute stroke care. To avoid counting suspected strokes that were not confirmed, we excluded all patients who spent one day or less in hospital and were discharged alive. If hospital admission criteria for patients with stroke changed over time, with an increasing threshold for admission, this might have artificially lowered the event rates. However, we found no evidence that any such clinical recommendations were introduced during the study years.

We could not extend our analysis to include data from more recent years, because NHS Digital, which now owns and distributes data that were in the custodianship of the Office for National Statistics, stopped supplying the full date of death and the month and year of birth from April 2012 onwards. Without a full date of death it is not possible to calculate 30 day case fatality; and the absence of information on date of birth meant that it is not possible to calculate age specific rates in more recent years than those covered by this study.

### Comparison with other studies

The Global Burden of Disease (GBD) study reported a stroke mortality rate for high income countries of 60.54 (95% confidence interval 57.21 to 67.00) per 100 000 in 2010, a close estimate to the rates reported in this study at 74 per 100 000 population in men and 72 per 100 000 population in women.^1^ The marginal difference in rates is a result of methodological differences between the two studies. For age standardisation, the GBD study used the WHO world standard population as their standard population, which results in about 20% lower estimates of the standardised rates than when using the European population, which was used in this study.^12^ The GBD study, studies comparing mortality trends in Europe, and researchers in England have produced consistent evidence on the reduction in stroke mortality rates between the 1990s and the early 2010s.^13^ ^14^ ^15^ The scale of reduction is similar to that reported in this study.

The stroke incidence rate in 2010 reported in the GBD study was 217 per 100 000, and as with the mortality rate, it is about 20-30% lower than our estimates of stroke event rates: 284 per 100 000 population in men and 234 per 100 000 in women. Choice of the standard population, and counting of multiple stroke events in our study, could explain the difference in results between the two studies.

In line with studies on trends in stroke incidence rates, we reported a reduction in stroke event rates over the study period.^13^ ^14^ However, our findings on age specific rates showed that the reduction was not universal: stroke rates declined in older people, no changes were observed in adults aged 20-34 years, and an increase of about 2% each year occurred in the age group 35-54 years. A stroke registry in south London reported similar findings on age specific rates.^16^ An increase in stroke incidence rates in young adults has been reported in France and America.^17^ ^18^

Published studies suggested that improvements in management of vascular risk factors have contributed to the observed reduction in overall stroke event rates.^19^ Others have emphasised the contribution of several stroke prevention strategies: hypertension control, use of statins, improved management of atrial fibrillation, reduction in smoking, decrease in salt consumption, and the introduction of nationwide clinics specialising in transient ischaemic attacks in England.^20^ ^21^ ^22^ ^23^

In contrast, stable or increasing stroke rates in younger age groups are likely to result from increasing rates of obesity and diabetes in younger people.^24^ ^25^ Substance misuse, including cocaine, has also been linked to increasing rates of stroke in young people.^26^ ^27^

We report on national trends in 30 day case fatality after stroke in specific age groups, as well as overall rates, over several individual calendar years. Our findings of a 40% reduction in case fatality between 2001 and 2010 are encouraging, and they are consistent with results of a study of primary care data, which analysed mortality at 56 days after stroke.^14^

Our estimates of age standardised case fatality of 44% in women and 42% in men in 2001, are higher than the case fatality reported in the MONICA study in the late 1980s.^7^ The higher rates in our study are mainly due to particularly high case fatality in older people; for example, 53% in men aged 85 years and older and 21% in men aged 55-64 years. In the MONICA study, which only included people younger than 65 years, case fatality in different centres ranged from 15% to 49% in men and 18% to 57% in women. After we restricted our analysis to people younger than 65 years, the case fatality decreased to 22% in men and 23% in women, comparable to rates reported in MONICA.

Our estimates of case fatality in 2001 at 22% in men and 23% in women, when age standardised to the 2013 European standard population, are comparable to the 20% case fatality reported for high income countries in a published review.^28^ The Oxford Vascular Study (OXVASC), conducted in Oxfordshire, overlaps with the early years of our study.^29^ The investigators reported case fatality of 17% in 2004, which is only moderately lower than the roughly 20% reported here. Our estimates are higher because they are combined estimates for the entire country, including the north of England, which historically has had higher stroke mortality than the southeast, where the data for OXVASC were collected.^29^

The contribution of specific aspects of the organisation of stroke care, medical and surgical interventions, and rehabilitation, to the observed reduction in case fatality needs to be further studied. However, the study period overlapped with a time of major changes to provision of stroke care nationally. These include unrestricted access to brain imaging; organisation of stroke units in all hospitals receiving patients with stroke; aspirin and thrombolysis for acute stroke; early supported discharge; and rehabilitation at home.^30^ ^31^ ^32^ ^33^

### Explaining factors behind observed reductions in stroke mortality

Our findings are consistent with the original MONICA study, for which data were collected between 1982 and 1995, and reported that two thirds of the decline in stroke mortality was attributable to a decline in case fatality and one third to a decline in event rates.^7^ Findings of the two studies are similar, despite being conducted in different times and in different populations—the MONICA study was undertaken years ago, did not cover England, and did not include people older than 64 years.

Although no studies have reported on determinants of reduction in stroke mortality rates in England, a study of myocardial infarction applied the WHO MONICA equation and used the same datasets and covered the same period as our study.^5^ It is well known that stroke and myocardial infarction share many common risk factors.^34^ ^35^ In contrast to our findings on stroke, the study of myocardial infarction reported that more than a half of the decline in mortality rates was due to a reduction in event rates and less than a half due to a reduction in case fatality. Thus, differences in the findings of the two studies, covering the same period and country, might suggest that prevention strategies were more effective in reducing the rates of myocardial infarction than of stroke. In contrast, acute care was more effective in improving short term survival of patients with stroke than of patients with myocardial infarction.

The observed variations in age specific trends in event rates between young and older people might reflect effective preventive strategies and policies, but ones with different levels of impact in different population subgroups. During the study years, prevention at the individual level was focused on reducing a 10 year risk of vascular disease.^36^ The implication of this approach was that middle aged and older people have been recognised as a high risk group, and therefore they are offered treatment to control their vascular risk factors. In contrast, younger people were categorised into an intermediate risk group and received no treatment, despite having higher life long risks of cardiovascular events. In 2014, the updated British consensus recommendations for the prevention of vascular diseases were published, which shifted the focus of prevention from short term risk to life time risk.^36^

### Implications for clinicians, policy makers, and researchers

Findings of the study showed that most of the reduction in stroke mortality is a result of improved survival of patients with stroke. However, acute and long term management of such patients is expensive, and the NHS is already spending about 5% of its budget on stroke care.^37^ By focusing on prevention and reducing the occurrence of stroke, major resources can be conserved.

The reported age specific trends provide important insights that can inform stroke prevention strategies. In 2009 the NHS started the Health Check programme aimed at reducing vascular disease risks and events in people aged 40-79 years. The evaluation of the first years of the programme showed suboptimal coverage, with only 10% of eligible people aged 40-59 attending clinics, but higher attendance rates among older people.^38^ The lack of interest in the NHS Health Check among younger adults, the group which, as found in this study, experienced an increase in stroke rates, highlights the importance of reviewing existing programmes or developing specific targeted interventions that appeal to this age group.

This study covered only one year after the NHS Health Check was introduced, and therefore rates reported here can be used as a point of reference against which to compare future studies of the population level impact of the programme. An updated analysis of the age specific rates of stroke events and case fatality, perhaps looking separately at intracerebral haemorrhage and cerebral ischaemia, as well as total stroke, might assess the impact of the NHS Health Check in different population groups. Younger adults need to be the focus of research to build the evidence base for effective and tailored interventions. Clinicians and policy makers should consider targeting risk factors for vascular disease in those aged 55 and younger, without compromising on achievements with prevention in older age groups.

Understanding trends in rates is necessary to compare changes in disease burden over time, but for clinicians, policy makers, and researchers, to plan resource allocation it is important to know if there are changes in absolute numbers of patients who require treatment. Our findings on no increase in the absolute numbers of stroke events and a reduction in stroke deaths are reassuring, and they are indicative of the reduction in stroke burden at population level.

### Unanswered questions and future research

Given that information on treatment and management of patients with stroke is limited in linked HES, further research using other data sources should explore the contribution of specific treatments and interventions to reduction in case fatality after stroke. Studies of changes in stroke severity over time could help to understand whether these had an impact on reduction in case fatality. A study of predictors of survival after stroke, including the impact of hyperacute care, centralised stroke care, and early rehabilitation on case fatality rates, would help in developing the optimal care path for patients with stroke. Analysis of data collected by the Sentinel Stroke National Audit Programme could provide answers. The increase in stroke events in younger people needs further exploration and monitoring. The risk factors that are contributing to the increase in stroke rates in people younger than 55 years need to be further investigated.

We did not report trends separately for haemorrhagic stroke and ischaemic stroke; other researchers might be able to do that if they have reliable diagnostic data on the types of stroke.

### Conclusions and policy implications

A marked decrease in stroke mortality rates observed in England in the first decade of the 21st century has been the result of improved survival of patients with stroke more than a decrease in event rates. Seventy two per cent of the reduction in mortality rates in men and 66% in women is attributed to a reduction in case fatality, and the rest to declines in event rates. The contribution of the two factors varied across age groups: in young adults all the reduction in mortality was due to decreases in case fatality, whereas among people aged 85 and older the reduction in case fatality and event rate contributed nearly equally. A reduction in case fatality occurred during the study years in all age groups. Reduction in stroke event rates in middle aged and elderly people, which resulted in the overall decrease in event rates, concealed the unfavourable trends in men and women younger than 55 years. This indicates that, although prevention was effective in reducing stroke event rates in older people, it failed in the young. Our findings show that improved survival of people with stroke is driving the reduction in stroke mortality. However, to reduce the burden of stroke care on hospitals and decrease the dependence on emergency services, prevention of vascular events needs to be strengthened, which would lead to reduction in stroke mortality through reduction in stroke occurrence.

**What is already known on this topic**

Stroke mortality rates have been declining in England, but little is understood about the factors influencing this decline
Previous studies have shown that stroke incidence rates have fallen in England, but there are conflicting results for trends in short term survival

**What this study adds**

Using linked hospital episode and mortality data, which include most of all stroke events in England, we found that age standardised stroke mortality rates halved between 2001 and 2010, and that this reduction was largely due to a reduction in case fatality, which decreased by 40%
The reduction in case fatality was observed in all age groups
Stroke event rates overall decreased by 20%, but this concealed an increase in event rates in people younger than 55 years

## Notes

Contributors: OOS conceived the study and is the guarantor. OOS, PS, and MJG developed the study design. MJG developed and led the building of the linked dataset. OOS undertook the data analysis and drafted the manuscript. All authors contributed to the interpretation of the data and revision of the manuscript. All authors had full access to all of the data in the study and can take responsibility for the integrity of the data and the accuracy of the data analysis. The corresponding author attests that all listed authors meet authorship criteria and that no others meeting the criteria have been omitted.

Ethical approval: Central and South Bristol research ethics committee (No 04/Q2006/176) approved the building and analysis of the linked dataset.

Data sharing: English mortality and hospital episode statistics can be obtained from NHS Digital at www.digital.nhs.uk.

Transparency: The manuscript’s guarantor (OOS) affirms that the manuscript is an honest, accurate, and transparent account of the study being reported; no important aspects of the study have been omitted; and there were no discrepancies from the study as planned.
